# Evaluating Tuberculosis and Drug Resistance in Serbia: A Ten-Year Experience from a Tertiary Center

**DOI:** 10.3390/antibiotics14030320

**Published:** 2025-03-18

**Authors:** Mihailo Stjepanovic, Snjezana Mijatovic, Nikola Nikolic, Nikola Maric, Goran Stevanovic, Ivan Soldatovic, Aleksandra Barac

**Affiliations:** 1 Clinic for Pulmonology, University Clinical Center of Serbia, 11000 Belgrade, Serbia; mihailo.stjepanovic@kcs.ac.rs (M.S.); snjezana.mijatovic96@gmail.com (S.M.); nikola.nikolic@kcs.ac.rs (N.N.); nikolamaric1994@gmail.com (N.M.); 2 Faculty of Medicine, University of Belgrade, 11000 Belgrade, Serbia; goran.stevanovic@med.bg.ac.rs; 3 Clinic for Infectious and Tropical Diseases, University Clinical Center of Serbia, 11000 Belgrade, Serbia; ivan.soldatovic@med.bg.ac.rs; 4 Department of Medical Statistics and Informatics, Medical Faculty, University of Belgrade, 11000 Belgrade, Serbia

**Keywords:** tuberculosis, latent tuberculosis, multidrug-resistance, treatment, Serbia

## Abstract

Background: Tuberculosis (TB) remains a leading cause of mortality worldwide, particularly in low- and middle-income countries. The rise of multidrug-resistant TB (MDR-TB) poses significant challenges to global health. This study reviews the experience of the largest pulmonology center in Serbia, a country with low MDR-TB incidence, focusing on TB prevalence, resistance detection, and treatment strategies between 2012 and 2021. Methods: We retrospectively analyzed a total of 1239 patients who were diagnosed and treated for TB in the period from 2012 to 2021 at University Clinical Center of Serbia. Results: Drug resistance was identified in 21 patients (1.7%), with the highest resistance to rifampicin (1.4%) and isoniazid (1.3%). Pyrazinamide and streptomycin resistance were detected in only a few cases. Patients with resistant TB were younger on average, though the difference was not statistically significant (46.4 ± 19.1 vs. 53.6 ± 18.4, *p* = 0.079). Prior TB history was more frequent in the resistant group, almost reaching statistical significance (4 vs. 82, *p* = 0.052). Conclusions: These findings underscore the critical importance of sustained surveillance, particularly of latent and drug-resistant TB forms, in alignment with the World Health Organization’s (WHO) TB control strategy to preserve Serbia’s low-incidence status. Moreover, given Serbia’s strategic location on a major migration route, there is an elevated risk of new TB cases emerging and potential shifts in TB-drug-resistance patterns developing in the future.

## 1. Introduction

Tuberculosis (TB) continues to represent a major global health burden, with approximately 1.3 million deaths reported in 2022 alone, making it the second leading cause of death from an infectious disease after COVID-19 [1], with a tendency to become the leading cause of death from an infectious disease, when combined with HIV, in the post-COVID era [2]. The incidence of TB in Serbia has a downward trend, and Serbia is considered a low-incidence country. In 2023, incidence in Serbia was 14 cases per 100,000, in Europe, 24 cases per 100,000, and globally 134 cases per 100,000 [3]. While TB predominantly affects the lungs, it can involve any organ system, contributing to its broad clinical spectrum. Transmission occurs primarily through inhalation of *Mycobacterium tuberculosis* aerosols from individuals with active TB [4,5].

Primary TB infection is usually accompanied by developing latent TB infection (LTBI). Approximately one-quarter of the global population is infected with LTBI, though only 5–10% will progress to active disease, typically within five years of primary infection [6,7,8]. Key risk factors for TB progression include HIV infection, diabetes mellitus (DM), malnutrition, smoking, and immunosuppression [9,10,11,12,13,14,15,16,17]. The incidence of TB is highest in low- and middle-income countries, with Asia and Africa bearing the greatest burden [18,19]. Reference methods for diagnosing active TB are direct microscopic seeking of bacillus in human samples, culture (the gold standard) and nucleic acid amplification tests (PCR–based procedures). The standard TB treatment consists of a regimen that includes combination of isoniazid (INH), rifampicin (RMP), ethambutol (EMB), and pyrazinamide (PZA), followed by a combination of INH and RMP only.

Although the struggle against TB resistances has been going on for over five decades, multidrug-resistant TB (MDR-TB) seems to be on the rise in recent years, with about 410,000 new multidrug-resistant/rifampicin-resistant TB (MDR/RR-TB) cases reported in 2022 globally, according to WHO. MDR-TB is a significant worldwide challenge due to resistance to key first-line drugs, RPM and INH, and often requires more complex treatment regimens.

The World Health Organization (WHO) End TB Strategy aims to reduce TB incidence by 90% and mortality by 95% by 2035 [1]. In this context, controlling drug-resistant forms of TB and improving the detection of latent TB are crucial. Considering that Serbia is a country with a low incidence of TB, so far, no research was conducted regarding the incidence of both TB and MDR-TB.

## 2. Results

### 2.1. General Epidemiological Trends

**Figure 1:**
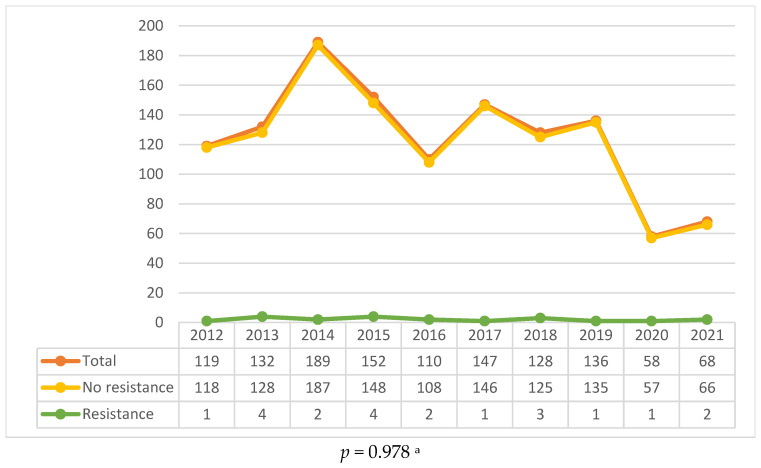
Distribution of patients by occurrence and by resistance to any/all TB drugs. ^a^ Chi-square test for trends.

From 2012 to 2021, a total of 1239 TB cases were reported at the Clinic of pulmonology, University Clinical Center of Serbia, with the highest annual incidence observed in 2014 (n = 189, 15.3%) and a marked decline in 2020 and 2021, coinciding with the COVID-19 pandemic (Figure 1). The decline in case numbers during the pandemic is consistent with global trends, where disruptions in healthcare services and diagnostic delays contributed to a temporary reduction in TB notifications.

### 2.2. Drug Resistance

Among the 1239 patients, drug resistance was identified in 21 cases (1.7%). Most common resistance was to RPM (n = 17, 1.4%), followed by INH (n = 16, 1.3%). Resistance to PZA and STM was detected in one and two patients, respectively. Notably, two patients were classified as having MDR-TB, defined as resistance to both RPM and INH. Table 1 provides a detailed breakdown of resistance patterns over the study period.

### 2.3. Patient Characteristics

Patients diagnosed with MDR-TB tended to be slightly younger, on average, at 46 years compared to 53 years for those with drug TB; however, this variance did not show significant statistical difference (*p* value = 0.079). Additionally, there was a prevalence of TB cases in the drug-resistant group, at 19%, almost reaching statistical significance (*p* value = 0.052). Moreover, individuals with a TB background were three times more likely to develop resistance compared to those without such a history (4.7% vs. 1.5%).

Pulmonary TB was the predominant form of the disease, affecting 87.2% of patients, while 11.7% had both pulmonary and extrapulmonary TB, and 1.1% had confirmed extrapulmonary TB. Among the extrapulmonary cases, pleural involvement was most common, affecting 65.1% of cases, followed by lymphadenitis, which accounted for 12.8%.

### 2.4. Comorbidities and Risk Factors

**Figure 2:**
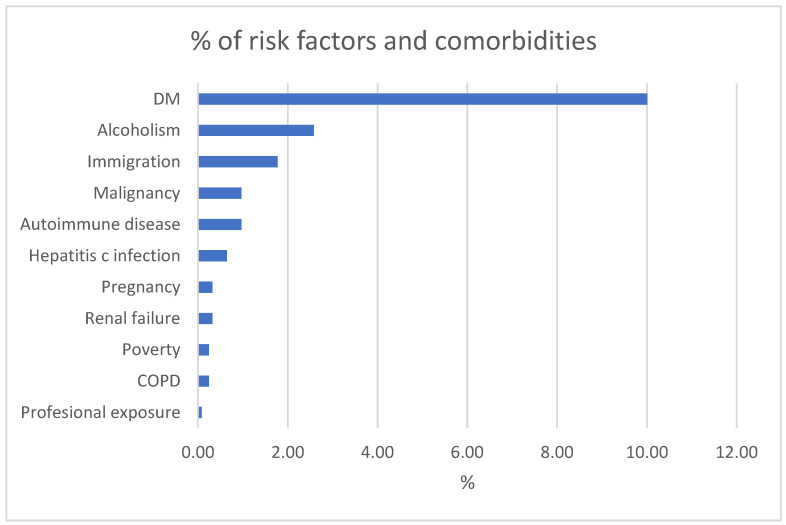
Risk factors for developing active tuberculosis (N).

DM was the most prevalent comorbidity, present in 55.1% of patients with comorbidities. This finding is consistent with studies showing that DM significantly increases the risk of active TB and worsens treatment outcomes. Alcoholism was the second most common risk factor, identified in 14.2% of cases (Figure 2).

## 3. Discussion

This paper provides significant details regarding the drug resistance and epidemiology of tuberculosis (TB) in a Serbian tertiary referral hospital. TB has been a severe public health issue for over ten years, and drug resistance makes constant vigilant monitoring essential. Our findings indicate drug resistance prevalence as 1.7%, corresponding to international and regional reports but also with room for additional MDR-TB dissemination, particularly among previously treated TB patients [1].

Though Serbia is generally a low-incidence nation for MDR-TB, our results on resistance percentage are noteworthy. Similar trends in other European nations such as Spain and Portugal are also seen with rising drug resistance, especially in patients with a history of prior TB treatment [20,21,22,23]. The relatively higher rate of resistance in our institution can similarly be attributed to its tertiary nature, as it is more likely to receive complex and drug-resistant cases [24]. This pattern follows international evidence, where referral health facilities with diagnostic service capacity for specialized cases tend to have concentrated MDR-TB cases.

Comparative studies within Balkan regions also corroborate these findings. For example, in Croatia, a five-year analysis of 2348 TB strains showed a rate of resistance of 3.69%, where isoniazid (INH) resistance was the most common, in contrast to the predominance of rifampicin (RPM) resistance in our study. Most notably, however, the Croatian research also identified genetic determinants of resistance, an aspect not yet a part of routine testing in Serbia [25]. Similarly, in Bulgaria, whole-genome sequencing (WGS) has also been used to follow the provenance and additional resistance profiles of RPM-resistant strains of TB, including fluoroquinolone, bedaquiline, and linezolid resistance [26].

Keeping these findings in view, the integration of genetic testing such as WGS into routine clinical evaluation of Serbian MDR-TB patients can yield more precise information regarding mechanisms of resistance. Due to the overall low TB incidence in the country, the selective application of WGS to complex cases could prove to be a cost-effective measure that would enhance patient management and inform national health policy for the future.

The WHO estimated that in 2022, approximately 410,000 people developed MDR-TB or RPM-resistant TB (RR-TB), with the highest burdens in countries such as India, China, and Russia [1,19,27,28]. Although Serbia does not fall within the WHO’s high-burden list, the increase in resistant cases, particularly among patients with a history of previous TB, highlights the importance of continued vigilance. In the majority of these patients, previous inadequate or incomplete treatment regimens have likely contributed to inducing resistance, as has been clearly outlined in the literature [29,30].

Previous TB treatment was more common among patients with MDR-TB, indicating a direct link between incomplete treatment and the acquisition of resistance. This result is also consistent with studies from other high-burden countries such as India and China, in which relapse cases and re-treatment due to incomplete treatment are significant causes of drug resistance; indeed, one study from India reported that retreatment patients had an almost four-fold higher risk of having MDR-TB compared with new cases [31].

The emergence of MDR-TB among those previously treated is multifactorial, and poor treatment adherence, limited access to drugs, and delays in diagnosis are all significant factors [32,33,34,35]. Although Serbia has made efforts to align the treatment of TB with WHO recommendations, our findings show that there is still room for improvement in the way individuals with a history of TB are handled. Enhancing patient management through personalized education, improved adherence monitoring, and early detection—as emphasized in the WHO End TB Strategy—would be capable of ensuring access to efficacious regimens and averting drug resistance development [1].

In our study, DM was the most prevalent comorbidity, occurring in 41.2% of patients with other illnesses. This finding is in accordance with international research, which indicates that DM not only increases the risk of active TB development by two- to three-fold, but also results in unfavorable clinical outcomes, including higher mortality [17,18,36,37,38]. This association was measured by a meta-analysis of Baker et al. (2011) which indicated heightened risk of TB among diabetic patients [39]. Furthermore, patients with poor control of blood sugar have more severe disease and take longer to recover. These results underscore the essential need for having combined management strategies for both TB and DM—such as routine screening for diabetes in TB patients and ensuring optimal glycemic control—to ultimately improve patient outcomes.

The interaction between DM and TB represents a major public health challenge in regions where both diseases are prevalent. Studies conducted in regions with high TB incidence, such as India and China, have found that DM not only increases susceptibility to TB but also complicates treatment outcomes [40,41,42]. In addition, patients with DM are at a higher risk of developing MDR-TB due to impaired immune responses and a higher likelihood of treatment complications [43]. The extremely high prevalence of diabetes mellitus (DM) in our patient population accentuates the urgency for joint control strategies for TB and DM in Serbia. Systematic screening of diabetics for TB and tailoring treatment regimens to individuals suffering from these two diseases may appreciably reduce the TB burden in this vulnerable group.

Another interesting observation from our study is the sudden drop of the TB cases in 2020 and 2021, coinciding with the COVID-19 pandemic. This is not a Serbia-specific trend; TB case notifications globally also fell, with the WHO reporting a 20% decline in 2020 compared to 2019 [44,45,46,47]. The misallocation of healthcare resources towards combating COVID-19, coupled with delayed diagnosis and disrupted treatment regimens, likely led to fewer reported TB cases [48]. The disruption may have prolonged impacts, as delayed diagnosis may amplify transmission among communities and potentially lead to a surge in new TB cases once healthcare systems return to normal. More focused post-pandemic recovery strategies, including catch-up screening and robust follow-up programs, will be necessary to mitigate these effects.

Several studies have underscored the risks posed by the COVID-19 pandemic to TB control efforts. For example, research in South Africa—a high-burden TB country—revealed a 50% drop in TB case notifications during the early months of the pandemic, mirroring the trends observed in our data [49,50]. This decline may lead to increased TB transmission and a higher prevalence of undiagnosed cases in the post-pandemic period. As healthcare systems recover, it is essential to re-establish robust TB screening and treatment programs.

The WHO End TB Strategy sets an ambitious goal to reduce TB incidence by 90% and TB-related deaths by 95% by 2035 [1]. Achieving these targets will require comprehensive strategies that extend beyond current diagnostic and treatment paradigms. Our study highlights the need for ongoing surveillance—especially for multidrug-resistant TB (MDR-TB)—and suggests that enhanced management of comorbidities, such as diabetes mellitus, could further improve TB control outcomes in Serbia.

In addition to refining clinical management, public health interventions must also focus on raising TB awareness among high-risk populations. Educational campaigns directed at individuals with diabetes, the elderly, and those with a history of TB treatment could promote early detection and improve treatment adherence, thereby reducing the risk of drug resistance.

Equally important is the expansion of diagnostic capacity. Rapid and accurate molecular diagnostic tools like GeneXpert must be made widely available in both urban and rural settings. Such accessibility is crucial for reducing MDR-TB transmission and ensuring timely, effective treatment.

It is important to acknowledge some limitations of our study. All patients were hospitalized to initiate TB treatment according to local guidelines, without initial stratification based on disease severity. To minimize this bias, we included all patients treated at our center, regardless of severity or drug resistance status. Once initial therapy was administered and no adverse events were noted, further treatment was conducted at regional centers or through the anti-tuberculosis dispensary, which precluded subsequent follow-up. Future research from other tertiary centers across Serbia is needed to obtain more consistent and comprehensive nationwide data, as well as to assess long-term outcomes.

## 4. Materials and Methods

### 4.1. Study Population

This retrospective study was conducted at the University Clinical Center of Serbia, the national referral hub for diagnosing and managing complex TB cases. We included 1239 patients, aged 16 to 97 years, who were diagnosed and treated for TB between 2012 and 2021. Patients were selected for TB testing based on clinical or radiological signs suggestive of the disease, or due to known contact with an infected individual. For patients unable to provide sputum samples, or for those with a high clinical suspicion of TB despite negative sputum cultures, bronchoscopy was performed to obtain bronchoalveolar aspirates. Data extracted from hospital records encompassed demographic information, the site of TB involvement, diagnostic criteria, prior TB history, any pulmonary or other comorbidities, and drug susceptibility profiles.

Importantly, no changes in diagnostic procedures were implemented during the study period, ensuring consistency across the dataset. The study was conducted in accordance with national ethical standards and received approval from the Ethics Committee of the University Clinical Center of Serbia (No. 524/11). The use of clinical medical data is approved from the Board of Clinic for Pulmonology, University Clinical Center of Serbia (No. 97, date: 17 January 2025).

### 4.2. Laboratory Methods

The biospecimens examined in this study were sputum, bronchoalveolar aspirate, blood, urine, cerebrospinal fluid, and tissue samples. *Mycobacterium tuberculosis* was identified using different methods: direct microscopy, molecular nucleic acid amplification (GeneXpert MTB/RIF) (Cepheid, Sunnyvale, CA, USA), liquid media system (BACTEC™ MGIT™ 960, Becton Dickinson Pty Ltd., Franklin Lakes, NJ, USA) for the *Mycobacterium* Growth Indicator Tube, and mycobacterial culture on Lowenstein-Jensen medium (Table 2). Most notably, all identified patients with drug resistance had positive culture for *Mycobacterium tuberculosis*, a measure that helped ensure the consistency of resistance data.

### 4.3. Resistance

TB drug resistance was conceptualized as resistance to one or more first-line anti-TB drugs. Monoresistance was identified when resistance was found against only one of the five first-line drugs: isoniazid (INH) (Bayer AG, Leverkusen, Germany), rifampicin (RMP) (Sanofi, Paris, France), streptomycin (STM) (Hangzhou Think Chemical Co., LTD., Hangzhou Zhejiang, China), ethambutol (EMB) (Sanofi, Paris, France), or pyrazinamide (PZA) (Sanofi, Paris, France). MDR-TB was identified as *Mycobacterium tuberculosis* strains that are resistant to both INH and RMP. All samples were subjected to drug susceptibility testing (DST) by standard protocols supported by the WHO, i.e., culture-based tests and the GeneXpert MTB/RIF assay (Cepheid, Sunnyvale, CA, USA) to screen for the principal first-line drugs: RMP, INH, PZA, and STM. These procedures ensure an exhaustive drug resistance assessment as per international practice for TB therapy.

### 4.4. Statistical Analyses

Statistical analyses were performed using SPSS software (version 29.0), and results were expressed as means ± standard deviation or medians for continuous variables and as counts and percentages for categorical variables. Comparative analyses between groups (MDR-TB vs. drug-susceptible TB) were performed using *t*-tests for continuous variables and Chi-square tests for categorical data. A *p* value < 0.05 was considered statistically significant.

## 5. Conclusions

In conclusion, our decade-long study at a Serbian tertiary center underscores that, despite a relatively low MDR-TB incidence, tuberculosis remains a significant concern, especially among previously treated patients and those with comorbidities like DM, while disruptions in TB notifications during the COVID-19 pandemic and Serbia’s strategic location on a major migration route heighten the risk of new cases and evolving drug resistance patterns. Correspondingly, long-term surveillance of drug-resistant and latent TB, coupled with better diagnostics and targeted public health interventions, is key to reducing the burden of TB and achieving WHO End TB targets.

## Figures and Tables

**Table 1:** Patients’ characteristics in total and by resistance.

Variable	Total	Resistance		
	N (%)	No (n = 1218)	Yes (n = 21)	*p* Value
Age	53.4 ± 18.4	53.6 ± 18.4	46.4 ± 19.1	0.079 ^a^
Form of TB				
Lung	1080 (87.2%)	1059 (86.9%)	21 (100%)	0.341 ^b^
Non-lung	14 (1.1%)	14 (1.1%)	0	
Both	145 (11.7%)	145 (11.9%)	0	
Previous TB	86 (6.9%)	82 (95.3%)	4 (4.7%)	0.052 ^b^
Age of previous TB	37.5 ± 12.6	37.8 ± 12.3	30.5 ± 17.3	0.256 ^a^
Sample				
Sputum	938 (75.8%)	920 (75.6%)	18 (85.7%)	0.165 ^b^
Aspirate	113 (9.1%)	110 (9.0%)	3 (14.3%)	
Biopsy	180 (14.5%)	180 (14.8%)	0	
Other	7 (0.6%)	7 (0.6%)	0	
Known contact	97 (7.8%)	95 (7.8%)	2 (9.5%)	0.677 ^b^
Previous lung disease	128 (10.3%)	124 (10.2%)	4 (19.0%)	0.263 ^b^
COPD, asthma	78 (60.9%)	76 (61.3%)	2 (50%)	0.643 ^b^
Carcinoma	33 (25.8%)	31 (25%)	2 (50%)	0.273 ^b^
Risk factors and comorbidities	296 (23.9%)	294 (24.1%)	2 (9.5%)	0.119 ^c^

^a^ independent samples T test; ^b^ Fisher’s Exact test; ^c^ Pearson Chi-square test; COPD—chronic obstructive pulmonary disease.

**Table 2:** Diagnostic methods for confirming acid-fasting bacilli/TB.

Methodology	Positive N (%)	Negative N (%)	Not Done N (%)
Direct microscopy	696 (56.2%)	532 (43%)	10 (0.8%)
GeneXpert *Mycobacterium tuberculosis*/rifampicin (MTB/RPM)	132 (10.7%)	9 (0.7%)	1097 (88.6%)
BACTEC™ MGIT™ 960	198 (16%)	7 (0.6%)	1033 (83.4%)
Lowenstein Jensen cultivation	1004 (81.1%)	172 (13.9%)	62 (5%)
Pathologic conformation	192 (15.5%)	12 (1%)	1034 (83.5%)
One diagnostic method	457 (36.9%)		
Two diagnostic methods	591 (47.7%)		
Three diagnostic methods	174 (14.0%)		
Four diagnostic methods	17 (1.4%)		

## Data Availability

The raw data supporting the conclusions of this article will be made available by the authors on request.

## Abbreviations

The following abbreviations are used in this manuscript:

DM	diabetes mellitus
DST	drug susceptibility testing
EMB	ethambutol
HIV	human immunodeficiency virus
INH	isoniazid
LTBI	latent tuberculosis infection
MDR/RR-TB	multidrug-resistant/rifampicin-resistant tuberculosis
MDR-TB	multidrug-resistant tuberculosis
MTB/RPM	*Mycobacterium tuberculosis*/Rifampicin
PZA	pyrazinamide
RMP	rifampicin
RR-TB	rifampicin-resistant tuberculosis
STM	streptomycin
TB	tuberculosis
WHO	World Health Organization
